# Reductions in biomarkers of exposure, impacts on smoking urge and assessment of product use and tolerability in adult smokers following partial or complete substitution of cigarettes with electronic cigarettes

**DOI:** 10.1186/s12889-016-3236-1

**Published:** 2016-07-11

**Authors:** Carl D. D’Ruiz, Donald W. Graff, Edward Robinson

**Affiliations:** Clinical Study Consultant, Greensboro, NC 27455-3407 USA; Celerion, Lincoln, Nebraska, USA; ITG Brands, Greensboro, NC USA

**Keywords:** Electronic cigarettes, Clinical trial, Biomarkers of exposure, Exclusive and dual use, Nicotine cessation, Urge to smoke, Adverse events

## Abstract

**Background:**

Electronic cigarettes (e-cigarettes) are popular alternatives to conventional cigarettes among adult smokers wishing to reduce their exposure to harmful smoke constituents. However, little information exists on the relative internal exposures resulting from the exclusive or dual use of e-cigarettes.

**Methods:**

Measurements of product use; adverse events; changes in smoking urge; and blood, urine and exhaled breath biomarkers of exposure (BoE) representing toxicants believed to contribute to smoking related diseases were made at baseline and after five days of product use in 105 clinically-confined smokers randomized into groups that partially or completely substituted their usual brand combustible cigarette with commercial e-cigarettes, or discontinued all nicotine and tobacco products.

**Results:**

Subjects switching to e-cigarettes had significantly lower levels (29 %–95 %) of urinary BoEs after 5 days. Nicotine equivalents declined by 25 %–40 %. Dual users who substituted half of their self-reported daily cigarette consumption with e-cigarettes experienced 7 %–38 % reductions, but had increases (1 %–20 %) in nicotine equivalents. Blood nicotine biomarker levels were lower in the cessation (75 %–96 %) and e-cigarette use groups (11 %–83 %); dual users had no significant reductions. All groups experienced significant decreases in exhaled CO (27 %–89 %). Exhaled NO increases (46 %–63 %) were observed in the cessation and e-cigarette use groups; dual users had minimal changes. By Day 5, all groups had greater reductions in smoking urge compared to cessation. However, reductions were larger in the dual use group. No serious adverse events were observed.

**Conclusions:**

Exposures to harmful smoke toxicants were observed to be lower in smokers who completely or partially replaced their cigarettes with e-cigarettes over five days.

## Background

Cigarette smoking is known to cause or exacerbate a number of chronic respiratory, neoplastic and cardiovascular diseases, which together account for approximately 480,000 premature deaths annually in the United States [1]. Although the combined efforts of governmental, educational and public health interests have reduced smoking to the lowest levels in recent decades, over 40 million Americans continue to smoke [2]. This reality suggests that novel approaches to reduce the burden of smoking-associated morbidity and mortality are needed. Electronic cigarettes (e-cigarettes) represent a rapidly-emerging product category that holds promise as a cigarette substitute that can simulate some of the familiar behavioral and sensory components of the smoking habit by generating an inhalable aerosol produced from mild electrical heating of a liquid of known composition. By eliminating the tobacco combustion processes that lead to the formation of many of the known harmful chemical constituents that are abundantly present in cigarette smoke, e-cigarette aerosols have a much simpler chemical composition which may result in less exposure to combustion cigarette smoke toxicants [3–8].

Recent scientific surveys and studies examining the habits and practices of e-cigarette users indicate that the dual use of cigarettes and e-cigarettes is common practice and that dual users believe e-cigarettes help reduce their consumption of cigarettes, health risks and family member exposure to secondhand combustible cigarette smoke [9–12]. Exposures to cigarette smoke constituents that result from dual use may be anticipated to depend on both the extent of daily uses of the respective products, as well as any conscious or subconscious changes in puffing topography consequent to any nicotine or sensory deficits that may derive from the partial substitution of cigarettes by e-cigarettes. Hecht et al. [13] had previously reported that reductions in daily cigarettes smoked resulted in reduced urinary excretion of NNAL, but those reductions fell short of those anticipated from the reduced numbers of cigarettes consumed. However, a more recent study of adult smokers who switched to using only e-cigarettes and to dual use of e-cigarettes and conventional cigarettes showed significant reductions in exposure to carbon monoxide and the toxicant acrolein over a four-week period [14].

The primary objective of this proof-of principal study was to compare changes in selected urine, blood and exhaled breath biomarkers of tobacco exposure among different groups of adult combustible cigarette smokers following a five-day forced-switch from usual brand conventional combustible cigarettes to exclusive use of e-cigarettes, dual-use of e-cigarettes and the subject’s usual brand combustible cigarette, or smoking cessation. The BoEs selected included a number of cigarette smoke constituents representing major classes of compounds believed to be the most significant contributors to smoking-associated disease risks. The secondary objectives of the study were to obtain estimates of daily product use and the amount of nicotine delivered (mg) following exclusive use of e-cigarettes or dual use of e-cigarettes and the subject’s usual brand combustible cigarette over a 5-day period; and assess the effectiveness of exclusive and dual use of e-cigarettes to reduce the urge to smoke in adult smokers.

## Methods

### Participants

The study protocol and the informed consent forms were approved by Chesapeake IRB, Columbia, MD. Two hundred fourteen potential subjects were recruited from the Lincoln, NE area using standard advertising methods (i.e., print and radio advertisements) and from a database of subjects who had previously participated in a clinical research study or who had expressed interest in participating in a study. All potential subjects were provided details regarding the study and written informed consent was obtained prior to initiation of any study procedures. One hundred two subjects were excluded for not meeting the predefined inclusion/exclusion criteria, while 15 subjects declined to participate prior to enrollment and two subjects were excluded because the study had reached the randomization target of 105 eligible subjects. The 105 subjects meeting the eligibility criteria were enrolled into the study and randomized into one of six study groups. Two subjects withdrew consent from the study following randomization for personal reasons unrelated to study participation. All subjects participating in the study were paid for their participation.

The main criteria for inclusion were as follows: healthy adult male and female smokers, 21 to 65 years of age, inclusive; smoker for at least 12 months and currently smoked an average of 10 or more king-size (100 mm in length) manufactured tobacco cigarettes per day; consistent use of their current usual brand style for 14 days prior to check-in; positive urine cotinine at screening (≥500 ng/mL); and exhaled carbon monoxide CO > 12 ppm at screening. Exclusion criteria included: history or presence of clinically significant mental or physical health conditions; females who were pregnant or breastfeeding; high blood pressure; body mass index < 18 kg/m^2^ or > 40 kg/m^2^; acute illnesses (e.g., upper respiratory infection, viral infection) requiring treatment within 2 weeks prior to check-in; use of prescription smoking cessation treatments, anti-diabetic or insulin drugs or medications known to interact with cytochrome P450 2A6; positive urine screen for alcohol or drugs of abuse; and self-reported puffers (i.e., smokers who draw smoke from the cigarette into the mouth and throat but do not inhale). Subjects who had used any tobacco- or nicotine-containing products other than manufactured tobacco cigarettes or e-cigarettes within 28 days of in-clinic product use were also excluded.

### Products tested

Three commercially available blu™ e-cigarette products were evaluated during this study: rechargeable tobacco flavor, rechargeable cherry flavor, and disposable cherry flavor. The rechargeable e-cigarettes consisted of a battery segment and a cartomizer segment comprising the heating unit and a liquid reservoir which can be separated from the battery for recharging or replacement when the e-liquid is depleted. The disposable e-cigarette was similar in form with the exception that the battery and cartomizer segments were included as a single, non-separable unit. Both units operated at a voltage of 3.7 volts (nominal). The resistance of the heating element was approximately 3 ohms for the disposable unit and about 3.5 ohms for the rechargeable unit. The maximum operating temperature of each unit was dependent on the charge level of the battery, the state of reservoir fluid fill, and on the manner of use and was not recorded in this study.

All e-cigarette products contained 24 mg/mL (2.4 %) USP grade nicotine, USP grade vegetable glycerin (~50 % in Cherry and ~80 % in Tobacco), USP grade propylene glycol (~45 % in Cherry and ~10 % in Tobacco), distilled water, and natural and artificial flavors. Both e-cigarette devices tested (rechargeable and disposable) utilized a closed tank system to contain the e-liquid, which had a volume of approximately 1.0 mL or 1.0 g by weight.

Subjects were provided unopened packs of their reported usual brand tobacco cigarettes for use during the study.

### Study design

This was a randomized, open-label, forced-switch parallel arm study conducted at a single independent research center (Celerion, Lincoln, NE) to assess biomarkers of tobacco exposure [15, 16] following short-term ad libitum use of electronic cigarettes by established adult smokers. This proof-of-concept study evaluated the hypothesis that use of e-cigarettes, either exclusively or with dual use of conventional tobacco cigarettes (with a 50 % reduction in self-reported CPD), can significantly reduce exposure to many of the chemicals commonly associated with use of combustible cigarettes in adult smokers. A cessation arm served as a maximum effect control group comparator. Further, evaluation under *ad libitum* product use conditions provided insights into product consumption and impacts on smoking urge under more natural use conditions.

Following successful screening and study qualification, subjects checked into the clinic on Day −2 and continued to smoke their usual brand cigarette *ad libitum* through the evening of Day −1. Subjects were confined in the research clinic for the entire duration of the study and a Fagerström Test for Cigarette Dependence (FTCD) was administered to all subjects upon enrollment. Baseline assessments occurred from the morning of Day −1 through the morning of Day 1 prior to the start of randomized product use and post-baseline assessments on the morning of Day 1 through the morning of Day 6.

On the morning of Day 1, subjects were randomized into one of six groups (*N =* 15 each):

Exclusive E-Cigarette Use Groups

- Group A1 – Tobacco flavor rechargeable e-cigarette
- Group A2 – Cherry flavor rechargeable e-cigarette
- Group A3 – Cherry flavor disposable e-cigarette

Dual Use Groups

- Group B1 – Tobacco flavor rechargeable e-cigarette + usual brand combustible cigarette
- Group B2 – Cherry flavor rechargeable e-cigarette + usual brand combustible cigarette
- Group B3 – Cherry flavor disposable e-cigarette + usual brand combustible cigarette

Cessation Group

- Group C – Complete nicotine cessation

### Product use

Use of the assigned products was documented daily and subjects were monitored during clinical confinement to ensure that no illicit nicotine or tobacco products were used. Subjects randomized to the cessation group were housed in an area of the clinic separate from the other groups. With limited exceptions, all product use was *ad libitum* from 07:30 to 23:00 on Days −2 to 5. These exceptions included meals, 15 min prior to blood sampling, and 30 min prior to exhaled CO and NO measurements.

None of the enrolled subjects reported previous use of e-cigarettes. As such, during enrolment, all subjects were trained on how to use the e-cigarettes and were also informed of how to notify the clinical staff of situations involving non-operating e-cigarettes. Subjects randomized to receive the e-cigarette products were allowed to carry them throughout the day in designated sections of the clinic. New e-cigarettes were supplied to the subjects each morning and throughout the day if the e-liquid solution was fully consumed or the product failed to work properly. All e-cigarettes were weighed before and after use.

Two levels of cigarette consumption reduction (100 % and 50 % from subject self-reports at Screening) were chosen to evaluate proof-of-concept, i.e., that replacing nicotine intake from a combustible cigarette with an e-cigarette may produce significant reductions in exposure to toxicants generated from the combustion of tobacco. Similar approaches have been utilized in prior studies evaluating the impacts of smoking reduction [13, 17]. Cigarette consumption was self-reported at Screening and subjects in the dual use group were required to reduce their daily cigarette consumption on Days 1–5 by ~50 % of that reported. Further, subjects randomized to the dual use group were required to request a cigarette product from the clinic staff and smoke only in specified sections of the clinic away from non-smoking subjects.

In order to assess how much nicotine was being delivered to the subjects, a rough estimate of the maximum amount of nicotine possibly delivered from each e-cigarette was calculated by utilizing a simple mass balance approach of multiplying the weight difference measured (before and after use) by 2.4 %. Each cigarette was assumed to deliver 1 mg of nicotine for the purpose of estimating the amount of nicotine administered. The total estimated amount of nicotine delivered per day for a subject (in mg) was the sum total of the estimated nicotine delivery for all e-cigarette units and the number of cigarettes smoked on each day. As a number of factors can contribute to nicotine uptake from e-cigarettes as well as combustible cigarettes (e.g., particle size, depth of inhalation, breath holding following inhalation), it is unlikely that the full volume of the e-cigarette solution indicated by the change in product weight before and after use, and thus the maximum possible amount of nicotine, was absorbed by the subjects. In the absence of a more precise method of estimating the actual dose of nicotine administered, the method used in this study was used to compare across study groups and should not be used to make a firm conclusion regarding nicotine uptake.

### Biomarker analysis

The urine and blood BoEs evaluated in this study (Table 1) were chosen to represent major classes of harmful and potentially-harmful cigarette smoke constituents that have previously been reported for cigarette smokers [1, 15, 16, 18–20]. All urine voided by each subject was collected in 24-h intervals from 07:30 on Day −1 through 07:30 on Day 1, and from 07:30 on Day 5 through 07:30 on Day 6, and aliquots were prepared from the 24-h collections. Blood samples were collected on Days −1 and 5 in the evening following dinner to assess exposure to CO and nicotine. Each biomarker was measured using validated methods based on: FDA’s Guidance to Industry for Bioanalytical Method Validation (2001); Good Laboratory Practices per 21 CFR Part 58; and the EMEA Guideline on Bioanalytical method validation (EMEA/CHMP/EWP/192217/2009 Rev. 1 Corr.2).

**Table 1:** Urine and blood biomarkers of exposure

	Chemical constituent	Clinical endpoint	Analysis method	LLOQ^b^
Urine biomarkers of exposure				
NNAL (4-(methylnitrosamino)-1–(3-pyridyl)-1-butanol)	4-(methylnitrosamino)-1–(3-pyridyl)-1-butanone (NNK)	Cancer	LC-MS-MS	5.00 pg/mL
3-HPMA 3-hydroxypropylmercapturic acid	Acrolein	Cancer	LC-MS-MS	20 ng/mL
HMPMA (3-hydroxy-1-ethylpropylmercapturic acid)	Crotonaldehyde	Cancer	LC-MS-MS	20 ng/mL
CEMA (2-cyanoethylmercapturic acid)	Acrylonitrile	Cancer	LC-MS-MS	0.275 ng/mL
1-OHP (1-hydroxypyrene)	Pyrene	Cancer	LC-MS-MS	10.0 pg/mL
NNN (N-Nitrosonornicotine)	NNN	Cancer	LC-MS-MS	0.200 pg/mL
MHBMA (Monohydroxy-3-butenyl mercapturic acid)	1,3-Butadiene	Cancer	LC-MS-MS	0.100 ng/mL
S-PMA (S-phenylmercapturic acid)	Benzene	Cancer	LC-MS-MS	0.0250 ng/mL
Nicotine equivalents^a^ Nicotine Nicotine-gluc Cotinine Cotinine-gluc Trans-3′-hydroxycotinine Trans-3′-hydroxycotinine-gluc	Nicotine	Nicotine exposure	LC-MS-MS	50.0 ng/mL 50.0 ng/mL 50.0 ng/mL 200 ng/mL 50.0 ng/mL 200 ng/mL
Blood Biomarkers of Exposure				
Blood COHb	CO	CO Exposure	Spectrophotometric	0.50 %
Plasma Nicotine	Nicotine	Nicotine Exposure	LC-MS-MS	0.200 ng/mL
Plasma Cotinine	Nicotine	Nicotine Exposure	LC-MS-MS	1.00 ng/mL
Plasma Trans-3′hydroxycotinine	Nicotine	Nicotine Exposure	LC-MS-MS	1.00 ng/mL

^a^Nicotine equivalents: calculated as the molar sum of nicotine and 5 major nicotine metabolites excreted in urine over 24 h and reported as nicotine equivalents (mg/24 h)

^b^ *LLOQ* Lower Limit of Quantification

### Exhaled breath biomarkers

Exhaled CO and NO are measures of acute carbon monoxide exposure and nitric oxide synthase activity, respectively. Smokers characteristically exhale higher CO [21] and lower NO [22] than do non-smokers. Exhaled CO and NO were measured during the study in the afternoon on Days −1 and 5 using a Bedfont Micro + Smokerlyzer and Niox Mino, respectively. Sampling was preceded by a 30-min (minimum) abstention from study product use.

### Urge to smoke

Urge to smoke was assessed in all subjects on Days −1 through 5 in the morning prior to the start of product use and in the evening using a simple and subjective 100 mm paper visual analog scale. VAS has been used in prior studies as a tool for measuring nicotine and smoking abstinence symptom suppression [23]. When using the VAS, participants were asked to rate “how strong is your urge to smoke right now?” by placing a cross through a 100 mm line where far left indicated: ‘not at all’ and far right indicated: ‘extremely’.

### Tolerability and adverse events (AEs)

Tolerability evaluations included assessments of AEs, vital signs and concomitant medications. AEs reported by the subjects or observed by the clinic staff were assessed for severity (mild, moderate, or severe), as serious or not serious, and relationship to the study products (unrelated, unlikely, possible, probable, or definite) by the Principal Investigator. A study product use-emergent AE was defined as an AE that started or intensified at the time of or after study product usage. For the cessation group, the AEs that occurred after randomization on Day 1 were treated as product use-emergent AEs.

### Determination of sample size

The sample size estimation was based on total NNAL because the group difference in percent change-from-baseline was expected to be smaller than the other biomarkers due to a longer half-life for elimination [24]. In a previous study, adult smokers who replaced cigarettes with a snus product or stopped all tobacco product use completely for 5 days excreted approximately 60 %–70 % less NNAL, while subjects who reduced cigarette use by half excreted approximately 30 % less total NNAL over the same timeframe [25]. Based on these results, a sample size of 12 was estimated to detect a 70 % reduction from baseline in the groups that stop smoking and to detect the differences between groups with at least 80 % power using two-sided testing. Up to 15 subjects were assigned to each group to ensure that a minimum of 12 subjects in each completed the study.

### Data analyses

Statistical analyses were performed using SAS procedures in SAS® Version 9.3. A paired *t*-test was used to make within-group comparisons between study days and a linear mixed model was used to assess between-group differences. Baseline values were included in the statistical models for the between-group comparisons as a covariate. Differences were considered statistically significant at an alpha level of 5 % and no adjustments were made for multiple comparisons.

In addition, regression analyses were performed for the Day −1 to Day 5 % change in urine biomarker concentrations against the Day −1 to Day 5 % change in CPD for the dual use groups and to evaluate the relationship between urine nicotine equivalents and the estimated amount of nicotine delivered by the e-cigarette products (Day 5 exclusive and dual use groups) and the number of cigarettes smoked (Day 5 in dual use groups, Day −1 in all groups).

## Results and Discussion

### Participant characteristics

A summary of the subjects’ demographics, tobacco product use history, and Fagerström Test for Cigarette Dependence (FTCD) scores [26, 27] for all study participants by study product sequence and overall is presented in Table 2.

**Table 2:** Summary of study demographics and FTCD scores by group and overall

	Exclusive e-Cigarette use groups			Dual use groups			Nicotine cessation *N =* 15	Overall *N =* 105
Trait/test	Tobacco rechargeable *N =* 15	Cherry rechargeable *N =* 15	Cherry disposable *N =* 15	Tobacco rechargeable *N =* 15	Cherry rechargeable *N =* 15	Cherry disposable *N =* 15		
Gender								
Female	6 (40 %)	3 (20 %)	9 (60 %)	6 (40 %)	3 (20 %)	7 (47 %)	3 (20 %)	37 (35 %)
Male	9 (60 %)	12 (80 %)	6 (40 %)	9 (60 %)	12 (80 %)	8 (53 %)	12 (80 %)	68 (65 %)
Race								
American Indian/Alaska Native	0 (0 %)	0 (0 %)	0 (0 %)	0 (0 %)	0 (0 %)	1 (7 %)	0 (0 %)	1 (1 %)
Black or African American	2 (13 %)	6 (40 %)	1 (7 %)	2 (13 %)	4 (27 %)	1 (7 %)	1 (7 %)	17 (16 %)
Black or African American, American Indian/Alaska	0 (0 %)	0 (0 %)	0 (0 %)	0 (0 %)	0 (0 %)	0 (0 %)	1 (7 %)	1 (1 %)
White	13 (87 %)	9 (60 %)	14 (93 %)	13 (87 %)	11 (73 %)	13 (87 %)	13 (87 %)	86 (82 %)
Age (years)								
Mean	37.1	40.1	33.9	36.6	36.8	39.3	41.1	37.8
SD	11.4	10.6	11.8	10.8	11.6	10.6	11.2	11.1
BMI (kg/m^2^)								
Mean	28.2	26.2	28.7	28.9	27.2	27.8	27.8	27.8
SD	5.5	6.5	5.7	5.5	4.7	5.1	4.9	5.4
Cigarettes per Day								
Mean	18.4	17.3	15.4	18.7	20.5	21.1	20.4	18.8
SD	7.1	6.2	3.3	6.6	7.3	5.8	7.5	6.5
Years Smoked								
Mean	19.2	20.3	15.0	19.3	14.6	21.7	21.3	18.8
SD	12.9	10.5	10.9	10.1	11.6	8.7	10.6	10.8
Usual Brand Cigarette Flavor								
Menthol	6 (40 %)	7 (47 %)	8 (53 %)	3 (20 %)	7 (47 %)	5 (33 %)	3 (20 %)	39 (37 %)
Non-Menthol	9 (60 %)	8 (53 %)	7 (47 %)	12 (80 %)	8 (53 %)	10 (67 %)	12 (80 %)	66 (63 %)
FTCD Score								
Mean	5.3	5.1	5.3	5.5	5.7	5.2	5.6	5.4
SD	1.5	2.0	1.5	2.0	1.1	1.7	2.0	1.7

### Product use

Table 3 summarizes the number of cigarettes (CPD) smoked by the different user groups (including CPD reported at Screening) and the estimated amount of nicotine delivered by the electronic cigarette products.

**Table 3:** Summary of the number of cigarettes smoked and estimated amount of nicotine delivered

		Exclusive e-Cigarette use			Dual use			Nicotine cessation
		Tobacco rechargeable	Cherry rechargeable	Cherry disposable	Tobacco rechargeable	Cherry rechargeable	Cherry disposable	-
Screening	Cigarettes	18.4 ± 7.1	17.3 ± 6.2	15.4 ± 3.3	18.7 ± 6.6	20.5 ± 7.3	21.1 ± 5.8	20.4 ± 7.5
Day −1	Cigarettes	16.9 ± 5.1	15.8 ± 5.4	14.9 ± 1.5	14.5 ± 4.5	14.2 ± 2.0	14.8 ± 2.4	17.5 ± 4.9
Day 1	Cigarettes	NA	NA	NA	8.9 ± 3.1	9.6 ± 2.7	9.8 ± 2.0	NA
	E-cigarette nicotine	14.9 ± 15.2	17.7 ± 16.9	13.6 ± 11.0	8.9 ± 6.9	4.6 ± 3.3	5.6 ± 3.4	NA
Day 2	Cigarettes	NA	NA	NA	8.9 ± 2.9	9.5 ± 2.8	9.9 ± 1.9	NA
	E-cigarette nicotine	17.2 ± 15.1	17.6 ± 14.3	19.6 ± 16.8	11.1 ± 7.8	6.5 ± 5.5	7.4 ± 7.2	NA
Day 3	Cigarettes	NA	NA	NA	8.9 ± 3.0	9.5 ± 2.5	10.1 ± 2.0	NA
	E-cigarette nicotine	16.3 ± 13.4	19.8 ± 16.9	19.4 ± 20.0	12.4 ± 9.5	6.2 ± 6.5	9.4 ± 9.8	NA
Day 4	Cigarettes	NA	NA	NA	9.0 ± 3.2	9.7 ± 2.9	10.0 ± 2.0	NA
	E-cigarette nicotine	18.4 ± 14.6	20.8 ± 14.2	23.4 ± 21.0	13.3 ± 10.7	7.5 ± 8.3	9.9 ± 9.1	NA
Day 5	Cigarettes	NA	NA	NA	9.0 ± 3.3	9.6 ± 2.8	10.4 ± 2.2	NA
	E-cigarette nicotine	19.4 ± 16.6	22.9 ± 16.6	22.9 ± 20.5	12.7 ± 11.6	7.0 ± 9.3	9.1 ± 8.6	NA
Day 1 through Day 5 Mean	Cigarettes	NA	NA	NA	44.7 ± 15.4	47.9 ± 13.6	50.5 ± 9.5	NA
	E-cigarette nicotine	86.1 ± 70.5	98.9 ± 75.1	99.0 ± 86.8	61.8 ± 45.9	31.9 ± 30.1	38.9 ± 36.4	NA

Values are presented as mean ± SD mg nicotine or cigarettes smoked

Cessation group subjects reported smoking 20.4 ± 7.5 CPD during screening and smoked 17.5 ± 4.9 cigarettes on Day −1

*NA* Not available

The mean reported cigarette consumption at Screening ranged from approximately 15 CPD to 21 CPD, with subjects to be randomized to exclusive use of the electronic cigarette products reporting fewer CPD than those randomized to the dual use and cessation groups. In order to standardize cigarette consumption during the study, subjects in the dual use groups were required to reduce their daily cigarette consumption on Days 1–5 by ~50 % from that reported at Screening. Indeed, subjects smoked ~52 % fewer cigarettes during the study compared to that reported at Screening. However, subjects tended to smoke fewer cigarettes during Day −1 compared to the number reported at Screening (exclusive e-cigarette use group ~0.5–1.5 fewer, dual user ~4–6.5 fewer, and the cessation group ~3 fewer). As a result, on Day 5 subjects in the dual use group reduced cigarette consumption by an average of ~33 % overall compared to Day −1 (individual range of ~ −64 % to +9 %).

On Day −1, mean cigarette consumption ranged from approximately 14 to 18 CPD with the subjects to be randomized to the dual use group tending to smoke fewer cigarettes compared to the other cohorts (Table 3). From Day 1 to Day 5, cigarette consumption within the dual use group was very consistent as subjects tended to smoke their entire daily allotment of cigarettes each day. Further, although the number of CPD was lower in the classic tobacco dual use group compared to the dual use cherry flavor use group, there were no statistically significant differences in Day 5 CPD or the change in CPD from Day −1 to Day 5.

Table 4 provides a statistical comparison of the estimated nicotine delivered on Day 5 between use groups.

**Table 4:** Statistical comparisons of the Day 5 estimated nicotine delivered by e-Cigarettes between use groups

Comparison	First LSM (mg)	Second LSM (mg)	Difference (mg)	*p*-Value
A1 vs B1	19.40	12.74	6.66	0.2140
A2 vs B2	22.86	7.04	15.83	***0.0038***
A3 vs B3	22.91	9.08	13.83	***0.0125***
A1 vs A2	19.40	22.86	−3.46	0.5170
A1 vs A3	19.40	22.91	−3.51	0.5117
A2 vs A3	22.86	22.91	−0.04	0.9934
B1 vs B2	12.74	7.04	5.70	0.2873
B1 vs B3	12.74	9.08	3.66	0.5011
B2 vs B3	7.04	9.08	−2.04	0.7074

*LSM* Least-square means

Use Groups

A1: Exclusive Tobacco flavor rechargeable e-cigarette

A2: Exclusive Cherry flavor rechargeable e-cigarette

A3: Exclusive Cherry flavor disposable e-cigarette

B1: Dual Tobacco flavor rechargeable e-cigarette and usual brand combustible cigarette

B2: Dual Cherry flavor rechargeable e-cigarette and usual brand combustible cigarette

B3: Dual Cherry flavor disposable e-cigarette and usual brand combustible cigarette

*p*-values indicate statistically significant

Use of e-cigarette products was roughly assessed as the estimated amount of nicotine delivered by the products as calculated from the difference in product weight before and after use and the percentage of nicotine in the products. The estimated nicotine intake from the e-cigarette products increased with time, peaking in each of the use groups on Day 4 or Day 5, but there was a great deal of variability in the amount of use within all of the groups as evidenced by the large SD values (Table 3).

On Day 5, the mean estimated amount of nicotine consumed by the exclusive use groups was very consistent, varying only by 3.5 mg across the use groups. Subjects using the tobacco flavored product received ~15 % less nicotine than those using any of the cherry flavored e-cigarette products, but the differences were not statistically significant. In contrast, the difference in product use among dual users was larger, varying by approximately 5.7 mg across use groups, with subjects using the tobacco flavored product receiving approximately 81 % and 40 % more nicotine than from the rechargeable and disposable cherry flavored products, respectively. However, these apparently large differences were not statistically significant (Table 4).

Predictably, subjects in the exclusive use groups used the e-cigarettes more on average than the subjects in the dual use groups, who were able to continue smoking. Use of the tobacco, cherry rechargeable, and cherry disposable by the exclusive use groups was ~52 %, ~225 %, and ~152 % more compared to the respective dual use groups, though the differences were statistically significant (*p* = 0.0038 and *p* = 0.0125) only for the cherry flavored products.

In addition, while there were no statistically significant differences among the exclusive use groups or among dual users on Day 5, over the course of the week, the tobacco flavor product tended to be used ~13 % less by the exclusive e-cigarette users compared to the two cherry flavored products, but nearly 2-fold more by the dual users compared to the cherry flavored e-cigarette products.

By making an assumption that dual users received ~1 mg of nicotine per cigarette, over the course of the entire week, it was calculated that subjects in the dual use tobacco, cherry rechargeable, and cherry disposable e-cigarette groups theoretically consumed ~107, ~80, and ~89 mg of nicotine, respectively. In comparison, the respective exclusive use groups theoretically received ~86, ~99, and ~99 mg of nicotine. As noted above, the interpretation of these estimates should be limited to the comparisons made in this study.

### Urine and blood biomarker comparisons

**Fig. 1:**
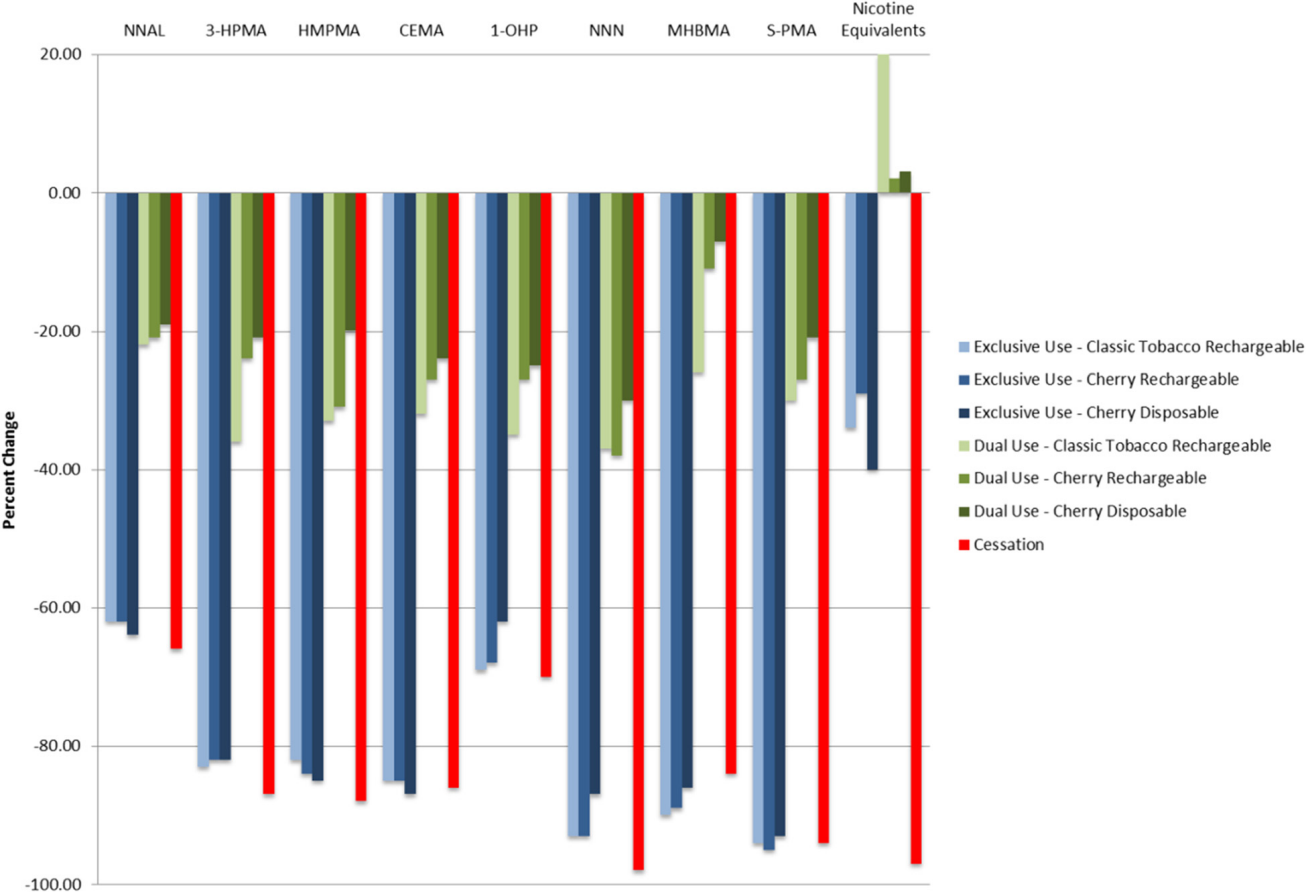
Urine biomarkers - Day 5 % change from Day −1

**Fig. 2:**
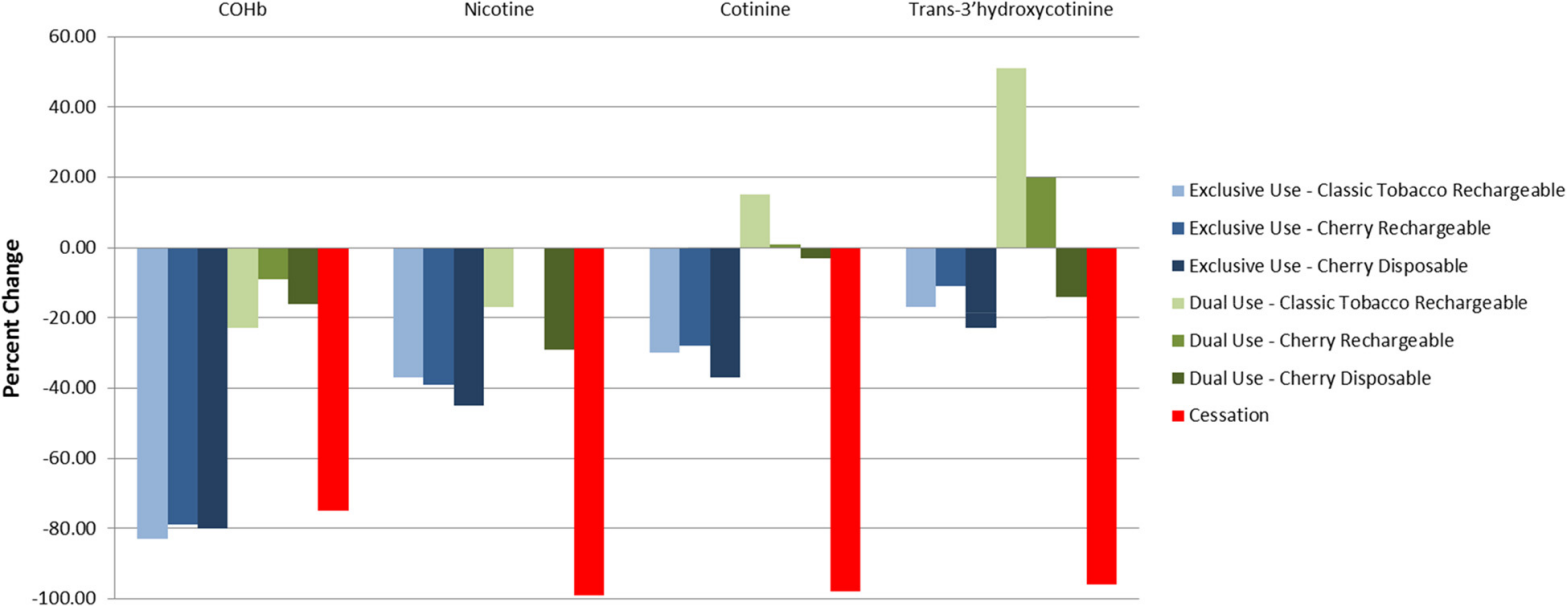
Blood biomarkers - Day 5 % change from Day −1

Reducing cigarette use for 5 days according to the requirements of the study tended to result in sizeable reductions in exposure to a number of known harmful biomarkers of tobacco exposure (Tables 5 and 6, Figs. 1 and 2). Smoking cessation lead to a 66 %–98 % reduction in excretion of the urine biomarkers of exposure evaluated in this study. The smallest reduction was seen in NNAL, which has the longest half-life of the individual biomarkers listed (approximately 45 days) [24]. Predictably, significant decreases were also observed in the COHb, nicotine, and the nicotine metabolites as the cessation subjects had no exposure to CO or nicotine.

**Table 5:** Urine biomarker concentration summary and statistical comparisons

	Exclusive e-Cigarette use groups			Dual use groups			Nicotine cessation *N =* 13
Biomarker	Tobacco rechargeable *N =* 15	Cherry rechargeable *N =* 13	Cherry disposable *N =* 13	Tobacco rechargeable *N =* 14	Cherry rechargeable *N =* 15	Cherry disposable *N =* 13	
NNAL (ng/24 h)							
Day −1	427.6 ± 218.8	383.7 ± 178.8	299.1 ± 165.0	430.8 ± 217.1	422.0 ± 257.5	343.3 ± 123.3	481.6 ± 377.5
Day 5	174.3 ± 144.6	149.2 ± 80.3	111.1 ± 68.9	328.6 ± 178.9	321.1 ± 177.3	269.2 ± 96.3	175.1 ± 140.8
p-value Day −1 vs Day 5	<0.0001	<0.0001	<0.0001	0.0063	0.0042	0.0028	0.0004
p-value Day 5 vs Cessation	0.1940	0.2456	0.2593	<0.0001	<0.0001	<0.0001	NA
3-HPMA (μg/24 h)							
Day −1	1521.7 ± 820.0	1903.0 ± 1132.7	1353.7 ± 598.8	1644.1 ± 501.3	1474.6 ± 519.9	1489.5 ± 567.1	2004.1 ± 1137.8
Day 5	214.4 ± 94.3	263.1 ± 64.7	246.7 ± 101.5	1046.2 ± 360.6	1070.7 ± 342.2	1155.4 ± 368.5	228.8 ± 84.2
p-value Day −1 vs Day 5	<0.0001	0.0001	<0.0001	<0.0001	0.0009	0.0062	<.0001
p-value Day 5 vs Cessation	0.5137	0.6099	0.3194	<0.0001	<0.0001	<0.0001	NA
HMPMA (μg/24 h)							
Day −1	523.8 ± 225.3	657.2 ± 328.9	533.4 ± 208.3	590.7 ± 178.7	597.5 ± 198.0	504.5 ± 167.1	797.7 ± 429.4
Day 5	71.3 ± 33.1	83.2 ± 32.3	78.0 ± 20.7	391.8 ± 151.2	394.6 ± 119.3	386.8 ± 94.1	78.1 ± 18.6
p-value Day −1 vs Day 5	<0.0001	<0.0001	<0.0001	0.0001	0.0001	0.0094	<.0001
p-value Day 5 vs Cessation	0.4785	0.5206	0.4211	<0.0001	<0.0001	<0.0001	NA
CEMA (μg/24 h)							
Day −1	219.7 ± 98.5	266.1 ± 140.9	201.0 ± 72.8	256.0 ± 97.9	246.2 ± 109.8	223.5 ± 61.6	289.7 ± 132.2
Day 5	33.4 ± 21.8	41.3 ± 30.4	25.9 ± 11.2	172.8 ± 72.1	168.3 ± 50.9	173.0 ± 63.7	41.0 ± 19.7
p-value Day −1 vs Day 5	<0.0001	<0.0001	<0.0001	0.0002	0.0010	0.0002	<.0001
p-value Day 5 vs Cessation	0.2902	0.6357	0.4549	<0.0001	<0.0001	<0.0001	NA
1-OHP (ng/24 h)							
Day −1	317.4 ± 138.7	302.9 ± 171.5	260.9 ± 166.8	363.6 ± 174.1	294.5 ± 145.5	304.1 ± 122.7	364.0 ± 200.7
Day 5	93.7 ± 52.9	85.9 ± 32.2	90.6 ± 38.4	235.1 ± 121.1	206.3 ± 90.9	224.1 ± 89.5	108.2 ± 55.0
p-value Day −1 vs Day 5	<.0001	0.0002	0.0007	0.0006	0.0004	0.0007	<.0001
p-value Day 5 vs Cessation	0.8331	0.7524	0.4115	<0.0001	<0.0001	<0.0001	NA
NNN (ng/24 h)							
Day −1	18.6 ± 12.1	13.7 ± 11.5	13.9 ± 12.5	14.3 ± 8.6	12.3 ± 8.1	11.3 ± 5.4	16.2 ± 12.1
Day 5	1.2 ± 2.4	0.7 ± 0.9	1.2 ± 3.3	8.9 ± 7.7	7.6 ± 5.4	7.1 ± 3.1	0.2 ± 0.1
p-value Day −1 vs Day 5	<.0001	0.0011	0.0045	0.0001	0.0019	0.0032	0.0005
p-value Day 5 vs Cessation	0.6402	0.6223	0.3974	<0.0001	<0.0001	<0.0001	NA
MHBMA (μg/24 h)							
Day −1	4.9 ± 3.5	5.9 ± 3.8	4.6 ± 3.2	5.0 ± 2.9	3.4 ± 2.5	4.5 ± 2.8	5.6 ± 3.6
Day 5	0.3 ± 0.1	0.3 ± 0.1	0.3 ± 0.1	3.5 ± 2.1	2.8 ± 1.8	4.3 ± 2.3	0.4 ± 0.1
p-value Day −1 vs Day 5	0.0002	0.0002	0.0006	0.0024	0.0320	0.1539	0.0002
p-value Day 5 vs Cessation	0.8548	0.8106	0.7313	<0.0001	<0.0001	<0.0001	NA
S-PMA (μg/24 h)							
Day −1	6.3 ± 3.7	8.1 ± 4.9	6.3 ± 3.9	7.0 ± 4.4	4.9 ± 3.5	6.9 ± 5.5	7.6 ± 4.3
Day 5	0.3 ± 0.1	0.3 ± 0.3	0.4 ± 0.2	4.9 ± 3.3	3.6 ± 2.3	6.0 ± 4.6	0.3 ± 0.2
p-value Day −1 vs Day 5	<.0001	<.0001	0.0001	0.0031	0.0055	0.0072	<.0001
p-value Day 5 vs Cessation	0.5274	0.7602	0.4430	<0.0001	<0.0001	<0.0001	NA
Nicotine Equivalents (mg/24 h)							
Day −1	17.0 ± 6.6	17.6 ± 8.7	14.5 ± 4.4	16.6 ± 5.5	16.0 ± 6.2	15.7 ± 3.9	20.0 ± 8.9
Day 5	10.7 ± 9.1	12.7 ± 9.7	10.5 ± 9.6	18.4 ± 7.2	15.9 ± 5.5	15.8 ± 5.0	0.5 ± 0.2
p-value Day −1 vs Day 5	0.0115	0.0415	0.0033	0.4188	0.9103	0.8519	<.0001
p-value Day 5 vs Cessation	<0.0001	<0.0001	<0.0001	<0.0001	<0.0001	<0.0001	NA

Values are presented as mean ± SD

**Table 6:** Blood biomarker concentration summary and statistical comparisons

	Exclusive e-Cigarette use groups			Dual use groups			Nicotine cessation *N =* 13
Biomarker	Tobacco rechargeable *N =* 15	Cherry rechargeable *N =* 13	Cherry disposable *N =* 13	Tobacco rechargeable *N =* 14	Cherry rechargeable *N =* 15	Cherry disposable *N =* 13	
Blood COHb (%)							
Day −1	6.3 ± 2.0	6.3 ± 2.0	6.0 ± 1.5	5.2 ± 2.0	4.8 ± 1.4	5.6 ± 1.9	7.4 ± 2.3
Day 5	1.2 ± 0.6	1.2 ± 0.5	1.0 ± 0.6	4.0 ± 1.5	3.8 ± 0.9	4.3 ± 1.3	1.6 ± 0.4
p-value Day −1 vs Day 5	0.0001	<0.0001	<0.0001	0.0179	0.0775	0.0170	0.0001
p-value Day 5 vs Cessation	0.5011	0.6009	0.4794	<0.0001	<0.0001	<0.0001	NA
Plasma Nicotine (ng/mL)							
Day −1	13.0 ± 6.1	14.7 ± 5.2	13.3 ± 6.5	12.5 ± 5.9	8.8 ± 3.2	11.1 ± 4.1	16.0 ± 7.0
Day 5	6.9 ± 6.3	8.4 ± 5.9	6.6 ± 5.6	9.5 ± 6.2	8.1 ± 5.2	7.9 ± 4.4	0.1 ± 0.0
p-value Day −1 vs Day 5	0.0033	0.0035	0.0053	0.0518	0.6197	0.0112	<0.0001
p-value Day 5 vs Cessation	<0.0001	<0.0001	0.0002	<0.0001	<0.0001	<0.0001	NA
Plasma Cotinine (ng/mL)							
Day −1	260.1 ± 128.1	299.9 ± 93.7	250.1 ± 92.4	247.6 ± 99.0	213.6 ± 62.8	218.4 ± 58.3	282.2 ± 135.9
Day 5	164.5 ± 167.4	202.1 ± 103.2	149.2 ± 116.1	261.5 ± 119.4	211.5 ± 70.2	212.9 ± 89.3	5.49 ± 6.7
p-value Day −1 vs Day 5	0.0438	0.0160	0.0112	0.6554	0.8935	0.7474	<0.0001
p-value Day 5 vs Cessation	<0.0001	<0.0001	<0.0001	<0.0001	<0.0001	<0.0001	NA
Plasma Trans-3′hydroxycotinine (ng/mL)							
Day −1	164.5 ± 167.4	202.1 ± 103.2	149.2 ± 116.1	261.5 ± 119.4	211.5 ± 70.2	212.9 ± 89.3	5.49 ± 6.7
Day 5	70.4 ± 59.0	85.0 ± 55.7	69.4 ± 56.5	102.2 ± 46.8	107.8 ± 50.7	98.5 ± 29.3	3.8 ± 2.7
p-value Day −1 vs Day 5	0.1626	0.3316	0.2073	0.0821	0.0051	0.1082	<0.0001
p-value Day 5 vs Cessation	<0.0001	<0.0001	0.0002	<0.0001	<0.0001	<0.0001	NA

Values are presented as mean ± SD

The changes from Day −1 observed in the exclusive use groups were mostly comparable to those seen in the cessation group, with the notable exceptions of the nicotine and nicotine metabolite urine and blood biomarkers as these subjects continued to receive nicotine from the e-cigarettes.

Dual users also tended to experience significant reductions in most of the biomarkers assessed, though to a lesser extent. Reductions in the urine BoEs for these groups ranged from ~20 % to 35 %.

With the exception of the nicotine and nicotine metabolite biomarkers, the Day 5 urine excretion and blood concentrations in the exclusive use groups were not significantly different from those of the cessation group. In contrast, the excretion and concentration of all biomarkers evaluated in this study were significantly higher in the dual use groups at Day 5 compared to the cessation group.

### Relationship between product use and urine biomarker excretion

Statistically significant, positive linear relationships were observed between percent change in biomarker excretion and the percent change in CPD smoked for all urine BoEs except nicotine equivalents (Tables 7 and 8). These results may indicate that smokers who reduce their cigarette consumption may predictably expect to see reduced exposure to a number of BoEs while replacing nicotine from combustible cigarettes with e-cigarettes.

**Table 7:** Regression analyses of nicotine equivalents excretion and Day −1 and Day 5 product use

Relationship Assessed	Slope	R-square	*P*-Value
Day −1 nicotine equivalents amount excreted against cigarettes smoked (all Groups)	0.7485	0.2137	<.0001
Day 5 nicotine equivalents amount excreted against cigarettes smoked (dual use Groups)	0.3598	0.0289	0.2814
Day 5 nicotine equivalents amount excreted against estimated nicotine from e-cigarettes (dual use Groups)	0.3970	0.4502	<.0001
Day 5 nicotine equivalents amount excreted against estimated nicotine from e-cigarettes (exclusive use Groups)	0.4794	0.8538	<.0001

**Table 8:** Regression analyses of the Day −1 to Day 5 % change in the amount of urine biomarker amount excreted against the % change in CPD

Urine Biomarker	Slope	R-square	*P*-Value
NNAL	0.4154	0.1518	0.0108
3-HPMA	0.6940	0.4105	<.0001
HMPMA	0.7878	0.4289	<.0001
CEMA	0.7096	0.4891	<.0001
1-OHP	0.6297	0.4227	<.0001
NNN	0.7766	0.3181	0.0001
MHBMA	0.7469	0.2874	0.0003
S-PMA	0.7259	0.4636	<.0001
Nicotine Equivalents	−0.0296	0.0002	0.9316

Further, a statistically significant, positive linear relationship (*p* < 0.0001) was observed between nicotine equivalents excretion and the number of cigarettes smoked on Day −1 when all groups were included, but not on Day 5 (*p* = 0.2814) when the dual use groups were included. Within the exclusive and dual use groups, the Day 5 relationship between nicotine equivalents excreted and the estimated nicotine from the e-cigarettes were statistically significant (*p <* 0.0001) and stronger than those with the number of cigarettes smoked. This contrasting finding could be due to the relatively consistent use and constant nicotine content in the e-cigarettes and the number of usual brand cigarettes smoked, coupled with differences in individual smoking behaviors.

### Exhaled breath

**Fig. 3:**
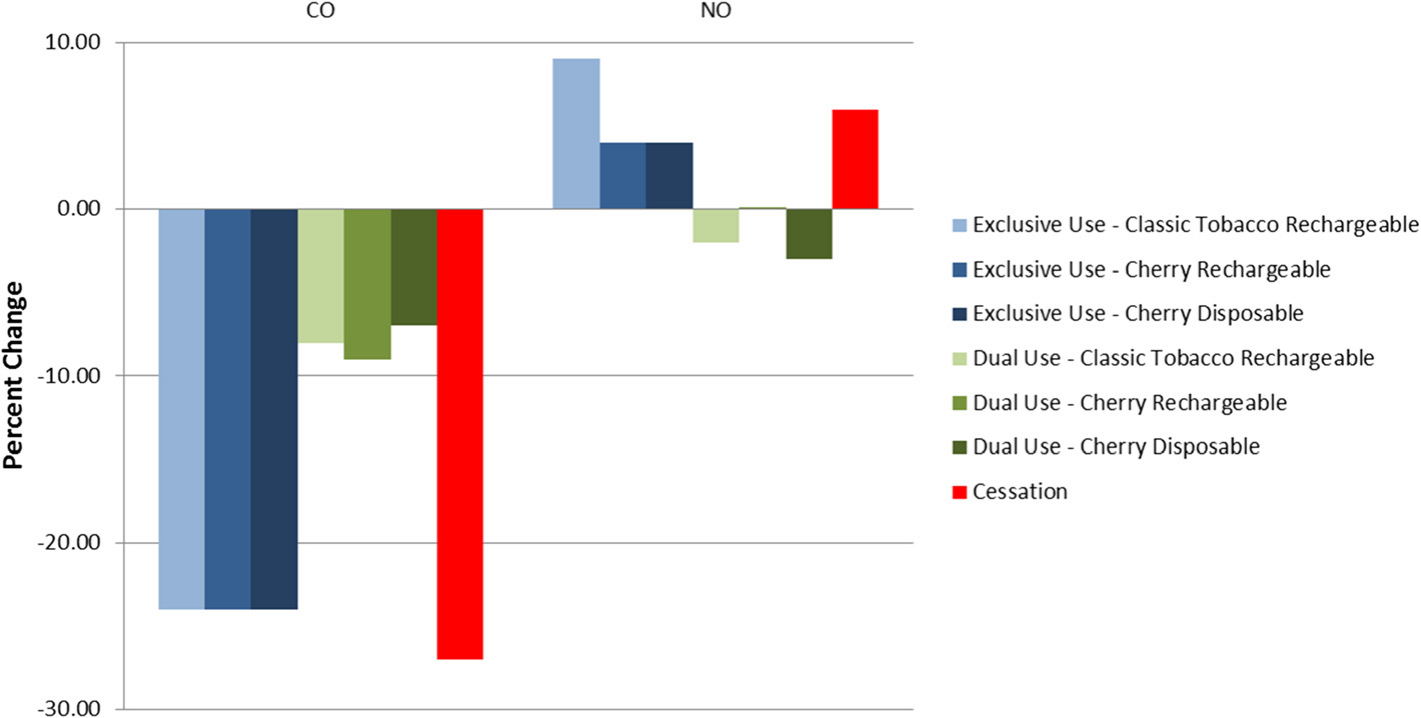
Exhaled Breath Biomarkers - Day 5 % Change from Day −1

Benefits associated with smoking reduction were noted in the exhaled CO and NO endpoints. All groups experienced statistically significant decreases in exhaled CO at Day 5 compared to Day −1, with decreases in the cessation and exclusive use groups ranging from ~88 % to ~89 % and in the dual use groups by ~26 % to ~32 % (Table 9 and Fig. 3). Further, there were no differences between the cessation and exclusive use group’s measurements on Day 5 whereas the dual use groups had significantly higher exhaled CO compared to cessation.

**Table 9:** Exhaled breath biomarker summary and statistical comparisons

	Exclusive e-Cigarette use groups			Dual use groups			Nicotine cessation *N =* 13
Biomarker	Tobacco rechargeable *N =* 15	Cherry rechargeable *N =* 13	Cherry disposable *N =* 13	Tobacco rechargeable *N =* 14	Cherry rechargeable *N =* 15	Cherry disposable *N =* 13	
CO (ppm)							
Day −1	27.2 ± 10.5	27.3 ± 6.9	26.9 ± 6.4	25.1 ± 7.3	25.4 ± 7.7	24.7 ± 5.5	29.3 ± 10.4
Day 5	2.9 ± 0.8	2.9 ± 0.8	2.7 ± 0.9	17.3 ± 5.7	16.1 ± 3.3	18.2 ± 5.7	2.8 ± 0.7
p-value Day −1 vs Day 5	<0.0001	<0.0001	<0.0001	0.0021	<0.0001	0.0002	<0.0001
p-value Day 5 vs Cessation	0.7990	0.8033	0.9109	<0.0001	<0.0001	<0.0001	NA
NO (ppb)							
Day −1	14.8 ± 12.8	11.5 ± 4.8	10.0 ± 4.0	14.9 ± 11.1	10.6 ± 4.6	14.3 ± 13.5	11.3 ± 4.0
Day 5	23.3 ± 21.6	15.5 ± 9.0	14.3 ± 6.5	12.9 ± 6.3	10.7 ± 4.4	11.4 ± 6.0	16.8 ± 10.1
p-value Day −1 vs Day 5	0.0075	0.1325	0.0053	0.3118	0.9415	0.3287	0.0321
p-value Day 5 vs Cessation	0.2370	0.6031	0.5674	0.0313	0.0615	0.0119	NA

Values are presented as mean ± SD

Exhaled NO was observed to increase from Day −1 to Day 5 in the cessation and exclusive use groups (~46 % to ~63 %) whereas the dual use groups experienced minimal changes. On Day 5, exhaled NO in the cessation and exclusive use groups was similar while and tended to be lower in the dual use groups, though not all comparisons were statistically significant.

### Urge to smoke

Table 10 provides a summary of the morning and evening urge to smoke findings and a statistical comparison within use groups. On Day −1, *ad libitum* use of the usual brand cigarettes resulted in mostly statistically significant reductions (*p* = 0.0001; < 0.0001 and 0.0325) in the urge to smoke from the morning assessment prior to product use to the evening assessment, with median percent reductions greater than ~39 % in 5 of the 7 product use groups. On Day 5, the median percent reduction tended to be larger in the dual use groups than the exclusive use groups, and the nicotine cessation group predictably had a small change in urge to smoke. However, only 1 of the 6 product use groups (the dual cherry flavored disposable e-cigarette use group) saw a median percent reduction of greater than 39 % and statistically significant reductions in the urge to smoke were seen only by the exclusive cherry flavored disposable (*p* = 0.0367) and the dual cherry flavored rechargeable and disposable e-cigarette use groups (*p* = 0.0019 and 0.0015, respectively).

**Table 10:** Urge to smoke summary and statistical comparisons within use groups

		Exclusive Use			Dual Use			Nicotine cessation
		Tobacco flavor rechargeable	Cherry flavor rechargeable	Cherry flavor disposable	Tobacco flavor rechargeable	Cherry flavor rechargeable	Cherry flavor disposable	
Day −1	Morning	71.4 ± 15.5	66.1 ± 24.8	58.3 ± 19.1	58.7 ± 27.3	61.1 ± 22.8	59.6 ± 23.0	58.5 ± 15.8
	Evening	33.6 ± 25.0	39.0 ± 25.1	44.3 ± 26.1	48.5 ± 29.4	32.2 ± 29.4	33.3 ± 25.5	35.6 ± 20.8
Day 5	Morning	73.9 ± 26.4	54.7 ± 39.7	66.6 ± 29.2	63.1 ± 25.9	65.5 ± 29.7	67.4 ± 25.0	65.1 ± 30.7
	Evening	61.1 ± 33.4	43.8 ± 35.4	52.7 ± 35.4	50.5 ± 28.9	35.1 ± 25.0	37.4 ± 27.5	66.0 ± 30.0
Day −1 Evening Change from Morning Urge to Smoke								
N		15	15	15	15	15	15	15
Mean ± SD		−37.8 ± 27.6	−27.1 ± 19.0	−14.1 ± 22.95	−10.2 ± 24.18	−28.9 ± 38.0	−26.3 ± 30.81	−22.9 ± 21.7
*p*-value		***0.0001***	***<0.0001***	***0.0325***	0.1247	***0.0106***	***0.0052***	***0.0011***
% change (median)		−53.2	−39.3	−17.9	−17.9	−58.3	−61.4	−48.6
Day 5 Evening Change from Morning Urge to Smoke								
N		15	15	15	15	15	14	13
Mean ± SD		−12.9 ± 30.3	−10.9 ± 24.4	−13.9 ± 23.3	−12.7 ± 27.8	−30.4 ± 30.8	−29.9 ± 28.0	2.5 ± 11.5
*p*-value		0.1227	0.1068	***0.0367***	0.0994	***0.0019***	***0.0015***	0.4563
% change (median)		−11.8	−1.0	−5.6	−26.1	−36.5	−44.3	1.2

Measured by Visual Analogue Scale

% change presented as medians due to outlier values that skew the means

*p*-values indicate statistically significant

Table 11 summarizes the Day 5 evening and Day 5 evening change from morning smoking urge statistical comparisons between use groups. The Day 5 evening urge to smoke was lower in all other groups compared to the nicotine cessation group, though only the comparisons to the exclusive cherry flavored rechargeable e-cigarette use group (*p* = 0.0355) and the dual cherry flavored rechargeable and disposable e-cigarette use groups (*p* = 0.0094 and 0.0229) were statistically significant. The exclusive tobacco flavored rechargeable e-cigarette groups did stand out amongst the product use groups, having an urge to smoke much higher than the rest, but there were no statistically significant differences between any of the product use groups.

**Table 11:** Day 5 evening and day 5 evening change from morning smoking urge statistical comparisons between use groups

Comparison	Day 5 evening comparisons				Day 5 evening change from morning comparisons			
	First LSM (%)	Second LSM (%)	Difference (%)	*p*-Value	First LSM (%)	Second LSM (%)	Difference (%)	*p*-Value
A1 vs C	63.00	66.32	−3.32	0.7589	−8.92	2.69	−11.61	0.2273
A2 vs C	43.32	66.32	−23.00	***0.0355***	−10.05	2.69	−12.75	0.1818
A3 vs C	49.91	66.32	−16.41	0.1323	−16.90	2.69	−19.59	***0.0427***
B1 vs C	45.74	66.32	−20.58	0.0611	−16.84	2.69	−19.53	***0.0443***
B2 vs C	37.69	66.32	−28.63	***0.0094***	−29.06	2.69	−31.75	***0.0012***
B3 vs C	40.90	66.32	−25.42	***0.0229***	−28.97	2.69	−31.66	***0.0014***
A1 vs B1	63.00	45.74	17.26	0.1111	−8.92	−16.84	7.92	0.4066
A2 vs B2	43.32	37.69	5.63	0.5971	−10.05	−29.06	19.00	***0.0401***
A3 vs B3	49.91	40.90	9.01	0.4108	−16.90	−28.97	12.07	0.2015
A1 vs A2	63.00	43.32	19.68	0.0668	−8.92	−10.05	1.14	0.9016
A1 vs A3	63.00	49.91	13.09	0.2226	−8.92	−16.90	7.98	0.3982
A2 vs A3	43.32	49.91	−6.58	0.5364	−10.05	−16.90	6.84	0.4594
B1 vs B2	45.74	37.69	8.05	0.4562	−16.84	−29.06	12.22	0.1921
B1 vs B3	45.74	40.90	4.84	0.6608	−16.84	−28.97	12.13	0.2019
B2 vs B3	37.69	40.90	−3.21	0.7664	−29.06	−28.97	−0.09	0.9921

*LSM* Least-square means

Use Groups

A1: Exclusive Tobacco flavor rechargeable e-cigarette

A2: Exclusive Cherry flavor rechargeable e-cigarette

A3: Exclusive Cherry flavor disposable e-cigarette

B1: Dual Classic Tobacco flavor rechargeable e-cigarette and a usual brand combustible cigarette

B2: Dual Cherry flavor rechargeable e-cigarette and a usual brand combustible cigarette

B3: Dual Cherry flavor disposable e-cigarette and a usual brand combustible cigarette

C: Nicotine cessation

*p*-values indicate statistically significant

As noted above, each of the product use groups experienced a reduction in the urge to smoke over the course of Day 5, while the nicotine cessation group experienced a slight increase. Statistically significant differences in the Day 5 evening change from morning urge to smoke compared to nicotine cessation were found with all three dual use groups (*p* = 0.0443, 0.0012 and 0.0014) and the exclusive cherry flavored disposable e-cigarette group (*p* = 0.0427). The only other statistically significant difference amongst the product use groups was found between the exclusive and dual cherry flavored rechargeable e-cigarette groups (*p* = 0.0401).

### Tolerability and adverse events

Overall, 72 mild product-use adverse events were experienced by 30 % of subjects. The number of subjects reporting adverse events ranged from 2 to 7 subjects each across groups receiving study products and only 1 subject in the cessation group. The most frequently reported adverse event was headache, reported 15 times by 12 subjects across study groups. There were no individual clinically significant laboratory and post product administration physical examination findings and or vital sign adverse events. Moreover, there were no serious adverse events and no subjects were discontinued due to adverse events.

## Conclusions

The results of this study demonstrate that smokers who completely substitute combustible cigarettes with e-cigarettes over a short period of time (5-days) experience reductions in exposure to a number of known harmful tobacco-related toxicants and carcinogens similar to smokers who quit smoking over the same period of time as measured by urine, blood and exhaled breath BoEs. As expected, the notable exceptions were the nicotine-related biomarkers, as subjects continued to consume nicotine in the e-cigarettes. The study also showed that subjects who switched to dual use also experienced significantly reduced exposure after partially replacing cigarettes with an e-cigarette product, albeit to a lesser extent.

Estimates of the theoretical amounts of nicotine delivered by the e-cigarette products used in this study were made based on the differences in product weights and nicotine content in order to provide context for comparisons across product use groups. However, the Day −1 to Day 5 changes in the maximum theoretical amount of nicotine delivered was not consistent with the reduction in the urine and blood nicotine biomarkers observed and suggests that far less than the maximum theoretical amount of nicotine from the e-cigarettes was available for uptake. Defining the “dose” of toxicants delivered by cigarette and e-cigarette products for systemic absorption is complicated by numerous consumer and product factors (e.g., inhalation depth and frequency, particle size, actual concentration in the e-liquid, battery strength, frequency of use) and indeed a large degree of variability in the estimated amount of nicotine delivered within each groups was observed. The estimated amount of nicotine delivered from the e-cigarette products increased with time but appeared to peak and became consistent by Day 5. Not surprisingly, subjects in the exclusive use groups used e-cigarettes on average more than subjects in the dual use groups, who continued to smoke conventional cigarettes. Further, the estimated theoretical amounts of nicotine delivered by the combustible and e-cigarette products used in both the dual and exclusive use groups suggests that the amounts of nicotine delivered were very similar, thereby suggesting that the exclusive use group was able to obtain similar levels of nicotine as the dual use group. However, this is an area for further research.

With regard to smoking urge, on Day −1, the *ad libitum* use of usual brand cigarettes resulted in mostly statistically significant reductions in the urge to smoke. By day 5, however, all groups had greater urge reduction compared to the nicotine cessation group, but the median percent reduction in urge to smoke tended to be larger in the dual use groups than the exclusive use groups. Furthermore, use of the e-cigarette products was well-tolerated and there were no serious adverse events observed under the conditions used in this study.

The study findings are consistent with an expectation of significantly reduced exposures to harmful smoke constituents in smokers who completely replace their cigarettes with e-cigarettes, and further with an expectation of potentially reduced risks for diseases believed to be caused by those exposures. Moreover, the study findings associated with exhaled breath biomarkers in the cessation and exclusive use groups were consistent with other research findings associated with reductions in exhaled CO and increases in NO following smoking cessation [28–35]; both of which are indicative of immediate and future health benefits.

The results of this study also support the findings of other investigations which have demonstrated that electronic cigarette use results in a decrease in certain biomarkers of tobacco exposure [15, 17, 36]. Furthermore, a recently-published study on e-cigarettes, including the brand evaluated in the present investigation, reported that the product’s emitted aerosol contained levels of harmful or potentially harmful constituents (HPHCs) such as carbonyl compounds, tobacco-specific nitrosamines, polycyclic aromatic hydrocarbons and other constituents that were on the order of 1500 times lower than those found in the smoke of conventional tobacco cigarettes (<2 μg/puff vs. ~3,000 μg/puff) [8]. The present study extends those findings with the observation that the e-cigarette produced markedly lower levels of exposure biomarkers when used by smokers in lieu of their preferred cigarette brand style for a period of 5 days.

In addition, the present investigation also further confirms and extends the findings of several prior, smaller studies that have compared levels of BoEs in users of e-cigarettes to those of conventional cigarette smokers. Vansickel et al. [37] and Farsalinos et al. [38] reported moderate plasma nicotine values in users of first generation e-cigarette devices similar to those used in the present study. Typical use of later-generation, tank-style e-cigarettes or intensive use of the first-generation cigarette-like devices has been reported to produce plasma nicotine values similar to those from conventional tobacco cigarettes [24, 39]. Hecht et al. [40] also have recently reported combined findings from three independent studies of smokers whose biomarkers levels were compared to those of 28 self-reported users of a variety of commercial cartridge- and tank-based e-cigarettes under uncontrolled *ad libitum* conditions. These authors concluded, with respect to the biomarkers analyzed, that the e-cigarettes had a more favorable toxicity profile than conventional tobacco cigarettes.

Dual use of electronic cigarettes and conventional cigarettes has been cited as a potential public health concern because of a possibility that it may expose smokers to greater health risks than those encountered by smoking combustible cigarettes alone [41]. The present study enforced a reduction in daily cigarettes smoked on the dual use group as an initial examination of the responsiveness of the measured smoke exposure biomarkers to moderately-reduced smoking combined with unlimited *ad lib* usage of e-cigarettes. Under these conditions, the study showed that Dual users’ experienced significant reductions in most of the biomarkers assessed (~20 % to 35 % reduction in urine biomarkers) and that the magnitude of reduction in exposure to BoEs in the dual use subject was roughly proportional to the reduction of combustible cigarettes smoked. While reducing the number of cigarettes smoked per day for 5 days is not sufficient to demonstrate a health benefit, the reduction in toxicants seen in the dual use subjects in this study may provide biological plausibility to previous long-term studies that have shown the potential for improving health outcomes by reducing cigarette consumption without complete cessation.

Further, the study findings associated with urge to smoke were encouraging as both the exclusive and dual use of e-cigarettes tended to result in greater urge reductions than cessation alone, though perhaps to a lesser extent than that achieved by smoking usual brand combustible cigarettes during the short term when smokers begin to switch to e-cigarette products. However, longer-term studies are needed to assess whether the exclusive use of e-cigarettes can result in consistent and statistically significant reductions in the urge to smoke.

Whether the reductions in toxic and carcinogenic smoke constituent exposure such as those observed in the present study may have the potential to similarly reduce risks for chronic, smoking-caused diseases for long-term e-cigarette users who have partially or completely abandoned cigarette smoking remains to be determined by epidemiologic or other types of investigations involving long-term use populations.

## Abbreviations

1-OHP, 1-hydroxypyrene; 3-HPMA, 3-hydroxypropylmercapturic acid; *ad lib*, *Ad lib*itum; AE, adverse event; BoE, biomarkers of exposure; CEMA, 2-cyanoethylmercapturic acid; CFR, code of federal regulation; CO, carbon monoxide; CoHb, carboxyhemoglobin; CPD, cigarettes per day; CRO, contract research organization; e-cigarettes, electronic cigarettes; EMEA, European Medicines Agency; FDA, food and drug administration; FTCD, fagerström test for cigarette dependence; g, gram; GLP, good laboratory practices; HMPMA, 3-hydroxy-1-ethylpropylmercapturic acid; HPHC, harmful or potentially harmful constituents; IRB – institutional review board; Kg, kilogram; LC-MS/MS, liquid chromatography-tandem mass spectrometry; LLOQ, lower limit of quantification; LSM, least-squares means; mg, milligram; MHBMA, monohydroxy-3-butenyl mercapturic acid; mL, milliliter; mm, millimeter; ng, nanogram; NNAL, (4-(methylnitrosamino)-1-(3-pyridyl)-1-butanol); NNN, N-Nitrosonornicotine; NO, nitric oxide; PG, propylene glycol; ppm, parts per million; SD, standard deviation; S-PMA, S-phenylmercapturic acid; USP, United States Pharmacopeia; VAS, visual analog scale
